# 3D-Printed Hydrogel Nanoplatforms for Sustainable Pest Control: Encapsulated Essential Oils as Biopesticides against *Bemisia tabaci*

**DOI:** 10.1021/acsomega.5c09494

**Published:** 2026-02-03

**Authors:** Raphaella Beatriz Barison Secco, Gabriela Patrícia Unigarro Villarreal, Felipe Franco de Oliveira, Juliana Milagres, Jhones Luiz de Oliveira, Daniele Ribeiro de Araujo, Leonardo Fernandes Fraceto

**Affiliations:** † Institute of Science and Technology of Sorocaba, São Paulo State University (UNESP), Sorocaba, São Paulo 18087-180, Brazil; ‡ B.Nano Technological Solutions LTDA, St Dr. Julio Prestes, São Miguel Arcanjo, São Paulo 18230-000, Brazil; § Centro de Ciências Naturais E Humanas (CCNH), 28105 Universidade Federal Do ABC (UFABC), Campus Santo André, São Paulo 09210-580, Brazil; ∥ 5758 The Connecticut Agricultural Experiment Station (CAES), New Haven, Connecticut 06511, United States

## Abstract

The overuse of synthetic pesticides has raised significant concerns owing to their adverse environmental and health effects, fostering interest in sustainable alternatives for pest management. Essential oils have emerged as attractive biopesticide candidates because they offer eco-friendly insect control solutions. In this study, we developed 3D-printed hydrogel prototypes that combined sodium alginate, pectin, and Pluronic F127 with slow-release systems of geraniol and eugenol encapsulated in zein nanoparticles. The nanoparticles exhibited high encapsulation efficiency (>99%), with an average diameter of 318 ± 28 nm, a polydispersity index of 0.41 ± 0.05, and a zeta potential of 29 ± 2 mV, and remained stable for over 60 days. The printed hydrogel prototypes exhibited homogeneous structures and good mechanical stability. Biological assays with *Bemisia tabaci* revealed a significant attraction effect, particularly for the pectin-based prototypes, with attraction rates exceeding 50%. These findings demonstrate the potential of nanoencapsulated essential oils in 3D-printed devices as efficient attractants, contributing to the development of sustainable tools for integrated pest management programs.

## Graphical abstract

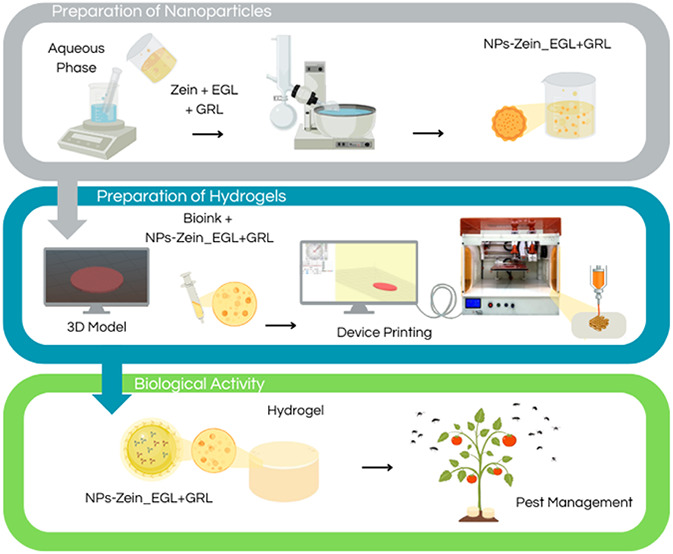

## 1 Introduction

Agriculture plays a crucial role in society, as it is the primary activity responsible for food production and the foundation of the global economy. This sector can be subdivided into various types and management systems, each with distinct characteristics related to resource utilization and applied techniques.^1^

Agricultural pests, particularly insects, pose a significant threat to agricultural production and cause substantial economic damage. These pests reduce crop productivity, alter harvest quality, and act as vectors for plant diseases. A notable example is the whitefly (*Bemisia tabaci*), which is a common pest of several crops. This insect causes direct damage that compromises vegetative and reproductive development. In addition, whiteflies are vectors of several geminiviruses that are responsible for large outbreaks of viral diseases in crops, making them one of the most significant agricultural pests worldwide.^2^ Control of this pest is traditionally carried out through strategies such as traps, biological control, and, mainly, chemical insecticides.^3^

Although the use of synthetic insecticides has been effective in controlling pests, this method has generated a series of problems over time.^4^ The indiscriminate use of synthetic chemicals contributes to the development of insect resistance, increases environmental contamination and production costs, and results in the loss of productive agricultural areas. These practices pose substantial risks to human and animal health.^5^

In response to these challenges, alternative control methods that are effective, environmentally friendly, and sustainable have emerged. Biopesticides have emerged as promising alternatives to synthetic insecticides. They have gained increasing attention because they are derived from natural sources and present low environmental impact and public health risks.^6^

Essential oils, widely recognized for their antifungal, bactericidal, and virucidal properties, are essential components of biopesticide formulations. Notable examples are eugenol (2-methoxy-4-(2-propenyl)-phenol) and geraniol (2*E*-dimethylocta-2,6-dien-1-ol), both of which are widely found in flora and have compounds that exert various biological activities, including repellent and attractive properties for insects, the effects of which depend on the concentration of the compound and the type of pest.^7^

The benefits of botanical compounds over synthetic insecticides include their rapid degradation in the environment and high selectivity, which can help slow the emergence of resistance in target organisms and make them ideal for safe use in various practical applications.^8^ However, their use in agriculture is hindered by their susceptibility to light, humidity, temperature variations, and microbial degradation. Therefore, there is a need to develop formulations that enhance the stability and efficacy of natural compounds in different settings.^9^

The encapsulation of essential oils in nanoparticles has proven to be a promising solution. This process enables the sustained and gradual release of active ingredients over time, enhancing their efficacy and stability, reducing the amount of input required to produce the release systems, and consequently lowering costs.^10^ Recent studies have suggested that using zein, a protein extracted from corn, may be an effective alternative for encapsulating these bioactive compounds, offering greater control over their release.^11^

Considering that the essential oils eugenol and geraniol, when evaluated individually, have shown promising results in the management of the agricultural pest *Bemisia tabaci*,^7,26^ including prolonged effects, repellent properties, and the ability to alter the behavior of the species, it became relevant to investigate the combination of these compounds in the present study.

Thus, the botanical compounds were encapsulated in zein nanoparticles, since the incorporation of nanoformulations into biopolymer films has already demonstrated the potential to enhance the antimicrobial and antibacterial efficacy of biopesticides.^12^ Furthermore, biopolymers used in encapsulation have advantages over synthetic polymers, such as low toxicity, biocompatibility, biodegradability, and renewability.^13^

For example, sodium alginate is widely used in extrusion bioprinting, allowing the incorporation of bioactive molecules into the polymer matrix via electrostatic interactions.^14,15^ This characteristic makes it ideal for sustained-release systems.

Pectin, a plant-derived polysaccharide of plant origin, has emerged as an improved system for the loading and release of active ingredients. Hydrogels, which are polymeric materials that can absorb and retain water within their three-dimensional networks,^10^ have been intensively studied in the field of biomaterials and demonstrate great potential for several applications.^16^ Similarly, Pluronic F-127, a block copolymer, is widely used because of its excellent biocompatibility and ability to increase protein stability, functioning as an efficient reservoir for the controlled release of active substances.^17^

Finally, 3D printing has emerged as a groundbreaking technology utilizing biopolymers, enabling the efficient and cost-effective creation of controlled-release prototypes.^18^ Specifically, extrusion bioprinting allows the deposition of inks with high cell density and compatibility with various materials and viscosities, offering benefits in terms of cost, time, and performance.^14^

In the literature, it has been observed that nanotechnology together with botanical pesticides can be used in trap systems, showing promise for applications in the control and management of agricultural pests, as in the case of chitosan nanoparticles containing geraniol in a study by de Oliveira et al. (2018),^7^ which demonstrated a significant attraction effect for whiteflies. More broadly, there have been reports of the use of food and chromotropic attractants in insect management strategies using traps.^19^

In this context, combining nanotechnology-based encapsulation of essential oils with 3D printing of biomaterials offers an environmentally viable strategy for pest control, including whitefly *Bemisia tabaci*, in agricultural systems.

In this study, we developed 3D-printed hydrogel prototypes composed of natural biopolymers (pectin, Pluronic F-127, and sodium alginate) loaded with encapsulated botanical compounds (eugenol and geraniol). The objective of this study was to evaluate the effectiveness of these systems in sustainable pest management and identify the most promising alternative prototype among those tested.

## 2 Materials and Methods

The materials used were geraniol (GRL, ≥98% purity), eugenol (EGL, ≥98% purity), zein, pectin (PEC), Pluronic F-68, and Pluronic F-127 (PF127), and they were obtained from Sigma-Aldrich. Ethanol was purchased from LabSynth. Acetonitrile (HPLC grade) was obtained from J. T. Baker. Membrane filters (0.45 μm) and Microcon regenerated cellulose filtration units (30 kDa) were purchased from Millipore. Sodium alginate (SA) and calcium chloride (CaCl_2_) were purchased from Dinâmica Química Contemporânea.

### 2.1 Preparation of Nanoparticles and Hydrogel Prototypes

#### 2.1.1 Preparation of Nanoparticles

The antisolvent precipitation method, as described by Hu and McClements (2014),^20^ was used to prepare mixtures of essential oils containing zein nanoparticles (NP-Zein) with minor modifications. Thus, an organic phase of zein (2 wt %, w/v) was prepared and solubilized in a hydroethanolic solution (85% v/v) under stirring overnight. The aqueous phase was prepared using Pluronic F-68 (2% w/v, pH 4) with constant stirring until it was fully solubilized. The zein solution was purified by centrifugation (30 min at 4500 rpm), heat treatment (15 min at 75 °C), and filtration through a 0.45 μm membrane (Millipore). The particles were prepared by adding 600 mg of each active ingredient, eugenol or geraniol (2% w/v), to 10 mL of a zein solution. The zein solution was then quickly added to the Pluronic F-68 solution under magnetic stirring for 30 min and placed in a rotary evaporator to remove ethanol to a final volume of 30 mL. Only zein and surfactants were added to the control formulations without active ingredients.

#### 2.1.2 Preparation of dos Prototypes

These polymers were selected for the prototypes due to their malleability, which enables the preparation of hydrogels in different shapes and sizes.^7^ This choice was supported by empirical experiments involving different ink combinations, through which rheological properties, such as fluidity and consistency, were explored to optimize printing time. Accordingly, parameters such as the extrusion flow rate and printing speed were systematically adjusted to ensure continuous extrusion and dimensional stability of the prototypes.

The Genesis printer (3D Biotechnology Solutions) was used to develop the extrusion method. First, the 3D design was created using 3D Printer Slicer v5.0.3 software, and the G-code was exported to Pronterface v3 to print the prototypes. Two prototype models were developed as listed in Table 1 S.

The prototypes are divided into two groups. Those in the first group were left to dry without any additional process or treatment; those in the second group were cross-linked with CaCl_2_ (2% m/v) for ±40 min to form hydrogels. This differentiation aimed to determine how the physical, chemical, and structural characteristics, influenced by the use of CaCl_2_, affect nanoparticle adhesion and the sustained release of the active ingredients.

A series of tests were performed with different printing parameters for each ink to determine the most effective ones for achieving the best results, as shown in Table 2 S with the final print parameters.

### 2.2 Characterization of Nanoparticles and Hydrogel Prototypes

#### 2.2.1 Nanosystem Stability

The physicochemical stability of control zein nanoparticles (NPs-Zein) and zein nanoparticles with active ingredients (NPs-Zein_EGL+GRL) was evaluated in terms of hydrodynamic size (nanometer), polydispersity index (PDI), zeta potential (mV), and encapsulation efficiency (EE) for 0, 7, 15, 30, 45, and 60 days. The samples were analyzed for the mean and standard deviation with *n* = 3 at 25 °C, by the Zetasizer Nano ZS90 (Malvern) at a fixed angle of 90°, using dynamic light scattering spectroscopy with an output wavelength of 532 nm through the Zetasizer Nano v3.30 software.

#### 2.2.2 High-Performance Liquid Chromatography (HPLC)

The efficiencies of EE were determined by placing 400 μL of the active formulation in a microtube with a cellulose ultrafiltration filter (Millipore) and centrifuging it for 10 min at 10,000 rpm. A C18 Phenomenex Luna (150 × 4.6 mm, 3 μm) 100A (Allcrom) reversed-phase column was used for quantification, and the system was maintained at 25 °C. The mobile phase consisted of a water–acetonitrile mixture (1:1, v/v). The analyses were performed at a flow rate of 1.0 mL/min, with UV detection at 240 and 305 nm, using an injection volume of 100 μL. HPLC then quantified the sample. The concentration of the encapsulated active was calculated by subtracting the added concentration (considering 20,000 μg/mL as 100%) from the concentration obtained in the ultrafiltrate reading. Under these conditions, the analytical curve for geraniol was obtained (*y* = −0.26 + 4.16*x*, *R* ^2^ = 0.99613, detection limit of 0.268198 μg/mL, and quantification limit of 0.893993 μg/mL), and for eugenol (*y* = 0.55 + 7.46*x*, *R* ^2^ = 0.97443, detection limit of 0.071397 μg/mL, and quantification limit of 0.237991 μg/mL (Figure 5S)).

#### 2.2.3 Rheological Properties

The stability of the hydrogels was evaluated by determining their rheological properties using a Malvern Kinexus oscillatory rheometer (Malvern Instruments, United Kingdom). Each measurement was made using the cone–plate geometry with a 0.1 mm gap between the plate and the geometry. Furthermore, to verify the effect of the tip, all samples were tested in duplicate with and without the tip. The evaluation steps were as follows:

- Analysis of *G*′, *G*″, and viscosity as a function of temperature: to analyze the effect of temperature on the structuring of materials, the samples were subjected to temperature variations between 10 and 50 °C, at 1 Hz and a pressure of 1 Pa.
- Flow curve: the strength and stability of the samples were tested and measured in terms of tensile stress σ and viscosity η, by varying the shear rate γ̇ . The shear rate range is from 0 to 200 s^–1^, at 25 °C. The following power law was used to obtain the values of consistency *K* and spreadability index *n* (eq 1).^31,32^
1 $$\sigma =K\cdot {\mathrm{\gamma ̇}}^{n}$$

The interactions between the material components were tested using the Cross model,^20^ as described in eq 2:

2 $$\eta \left(\mathrm{\gamma ̇}\right)=\eta_{\infty }+\frac{\eta_{0}-\eta_{\infty }}{1+{\left(C\mathrm{\gamma ̇}\right)}^{m}}$$

where *η*_0_ is the shear viscosity when *γ̇* = 0, *η*_∞_ is the shear viscosity at infinity, *C* is the time constant, and *m* is the Cross index.

- Analysis of *G*′ and *G*″ as a function of shear (or sweep amplitude): the stability of materials when subjected to shear was studied by varying the shear between 0.001% and 200%, at 25 °C.

#### 2.2.4 Scanning Electron Microscopy (SEM) and Atomic Force Microscopy (AFM)

These microscopies were used to characterize the surface structure of the prototypes and nanoparticles as well as their composition and topology. For this purpose, JEOL JSM-6010 model equipment was used, and the samples were prepared by using a Sputter Set Point metallization of 30 mA with a process time of 60 s. The characteristics used were an SEI beam (secondary) with a voltage of 11 kV, a spot size (SS) of 30, and magnifications of 100×, 300×, 500×, and 1000×. The analysis was performed using an Easyscan 2 microscope (Nanosurf, Switzerland), operated in the noncontact mode with TapAl-G cantilevers (BudgetSensors, Bulgaria) at a scan rate of 90 Hz.

#### 2.2.5 Fourier Transform Infrared Spectroscopy (FTIR)

Structural characterization was conducted using Fourier Transform Infrared Spectroscopy (FTIR) on an Agilent Technologies Cary 630 spectrometer in Attenuated Total Reflectance (ATR) mode. Scans were performed at 128, with a nominal resolution of 4.0 cm^–1^, in the 4000–400 cm^–1^ range.

### 2.3 Biological Activity

#### 2.3.1 *Bemisia tabaci* MEAM1 Rearing

Adult *Bemisia tabaci* MEAM1 were obtained from the Plant Virology Laboratory, Department of Plant Pathology, University of São Paulo (USP/ESALQ), Piracicaba, Brazil. The colony was maintained on collard plants (*Brassica oleracea*) under controlled environmental conditions: 28 °C, 55% relative humidity, and a 14:10 h light:dark photoperiod. Mixed-age adult whiteflies of both sexes were used in all the assays.

#### 2.3.2 Two-Choice Bioassays: Device Preference Over Time

Two-choice arena bioassays were designed according to Schlaeger et al. (2018)^27^ with adaptations (Figure 1S). The experiments were performed in arenas constructed from plastic Parafilm-sealed Petri dishes connected to a 10 mm diameter plastic tube. The tube featured a central opening through which the insects were introduced and positioned 5 cm from each Petri dish, equidistantly. In each assay, 20 adult whiteflies were released into the central opening and allowed to choose between two stimuli placed individually in each dish.

The experiments were conducted under controlled environmental conditions: 24 °C, 60% relative humidity, and continuous light. Stimulus combinations varied across assays and included comparisons between the treatment and control devices, control devices and empty dishes, leaves and empty dishes, and empty and empty configurations.

The insects were observed at 15, 30, 60, 120, and 180 min intervals after release. The number of whiteflies that settled on each stimulus was recorded at each time point. Each stimulus combination was tested in triplicate.

#### 2.3.3 Two-Choice Bioassays: Leaf vs Device

A separate set of bioassays was conducted to evaluate whitefly preference between a detached tomato leaf (*Solanum lycopersicum*) and a synthetic device, either as a treatment or its corresponding control. The arena design and experimental conditions were consistent with those described previously. Each assay consisted of one leaf positioned in one dish and a device or control stimulus placed in the opposite dish.

Additional control treatments included leaf vs leaf (to assess innate side bias or stimulus symmetry) and empty vs leaf (to establish baseline attraction to the leaf alone). In all of the experiments, adult whiteflies were released at the center of the arena and allowed to respond for 180 min.

At the end of the exposure period, the number of insects settled on each stimulus was recorded. Each treatment was replicated three times using independent groups of insects.

#### 2.3.4 Statistical Analysis

For the characterization of nanoparticles, data analysis and graph generation were performed using OriginLab 8, including particle size distribution, PDI, zeta potential, and encapsulation efficiency. For morphological characterization of the 3D-printed prototypes, surface and morphology images were processed and analyzed using Gwyddion v2.0 software. Bioassay data analyses were conducted using R version 2025.05.0 Build 496 (R Core Team, 2025). In the time-course bioassays, statistical comparisons were focused on the final time point (180 min), where insect preferences were expected to stabilize. For each treatment pair, the total number of insects choosing each option across the three replicates (20 insects per replicate) was summed and analyzed using Pearson’s chi-square tests (2 × 2 contingency tables). Differences were considered statistically significant when *p* < 0.01 and are indicated by an asterisk (*) in the corresponding figure panels. In the leaf vs device bioassays, the proportion of insects selecting each option was calculated per replicate, expressed as mean ± standard error of the mean, and visualized using horizontal stacked bar plots. Data visualization and statistical plotting were performed using the ggplot2 package in R. No correction for multiple comparisons was applied, and all *p*-values were reported to facilitate interpretation.

## 3 Results and Discussion

### 3.1 Physicochemical Stability of the Nanoparticles

The physicochemical characterization of the control and active nanoparticles considered the following parameters: average size (nm), PDI, ZP (mV), pH, and EE (%) (Table 3S and Figure 2S).

Over the 60-day evaluation, the NPs-Zein exhibited a slight but consistent increase in size, remaining below 150 nm, and demonstrated an average hydrodynamic diameter of 128 ± 22 nm, whereas the NPs-Zein_EGL + GRL showed a gradual decrease in particle size from 318 ± 28 to approximately 280 nm by day 60 (Figure 2Sa). This size reduction could be associated with rearrangement or relaxation of the encapsulated device over time. Analogously, compared with images obtained by AFM, the analysis of the nanoparticles revealed a rounded shape with an average diameter of 300–450 nm, confirming the approximate average size recorded by dynamic light scattering spectroscopy. Compared with the literature, the average size of the nanoparticles is within the typical range for zein-based systems (10–500 nm).^22^

Regarding polydispersity (Figure 2Sb), the NPs-Zein maintained a low and stable PDI (0.16 ± 0.03), indicating homogeneous particle distribution throughout storage. In contrast, NPs-Zein_EGL + GRL initially had a higher PDI (0.43 ± 0.05), which decreased slightly over time, suggesting reduced particle-size variability and potential interactions between the oils and the polymer matrix. In other published studies, the PDI values for NPs-Zein are consistent with the expected range for monodisperse systems (0.13–0.19).^20^

Zeta potential values (Figure 2Sc) remained consistently above 25 mV for both formulations, which is the threshold generally associated with colloidal stability. NPs-Zein exhibited a minor decline in zeta potential over time (28.9 ± 3.9 mV), while NPs-Zein_EGL + GRL maintained or slightly improved their surface charge (30.3 ± 1.5 mV), reinforcing the good colloidal stability of the encapsulated systems during prolonged storage. This indicates good colloidal stability due to sufficient electrostatic repulsion and suggests reduced aggregation tendencies, as higher absolute values correlate with improved stability.^23^

Encapsulation efficiency was greater than 99% for NPs-Zein_EGL + GRL, demonstrating the effectiveness of zein as a nanocarrier for essential oils. The efficiency calculations were based on the calibration curves for both active compounds. The calibration curve equations for EGL and GRL were *y* = 7.46*x* + 0.55 (*R* ^2^ = 0.97443) and *y* = 4.16*x* – 0.26 (*R* ^2^ = 0.99036), respectively, as shown in Figure 5S.

These results confirmed the physicochemical stability of both nanoparticle systems over 60 days, with NPs-Zein_EGL + GRL showing an interesting trend of size reduction and a stable surface charge, which may positively influence their application as delivery systems for essential oils.

### 3.2 Physicochemical Stability of Hydrogels

The inks were successfully 3D-printed by using the parameters defined in Table 2S, following the sampling scheme illustrated in Figure 1. For each ink, two concentrations of active compounds were evaluated (300 mg of GRL + 358 mg of EGL, and 251 mg of GRL + 300 mg of EGL), along with two device formats: with and without CaCl_2_ cross-linking treatment.

**Figure 1:**
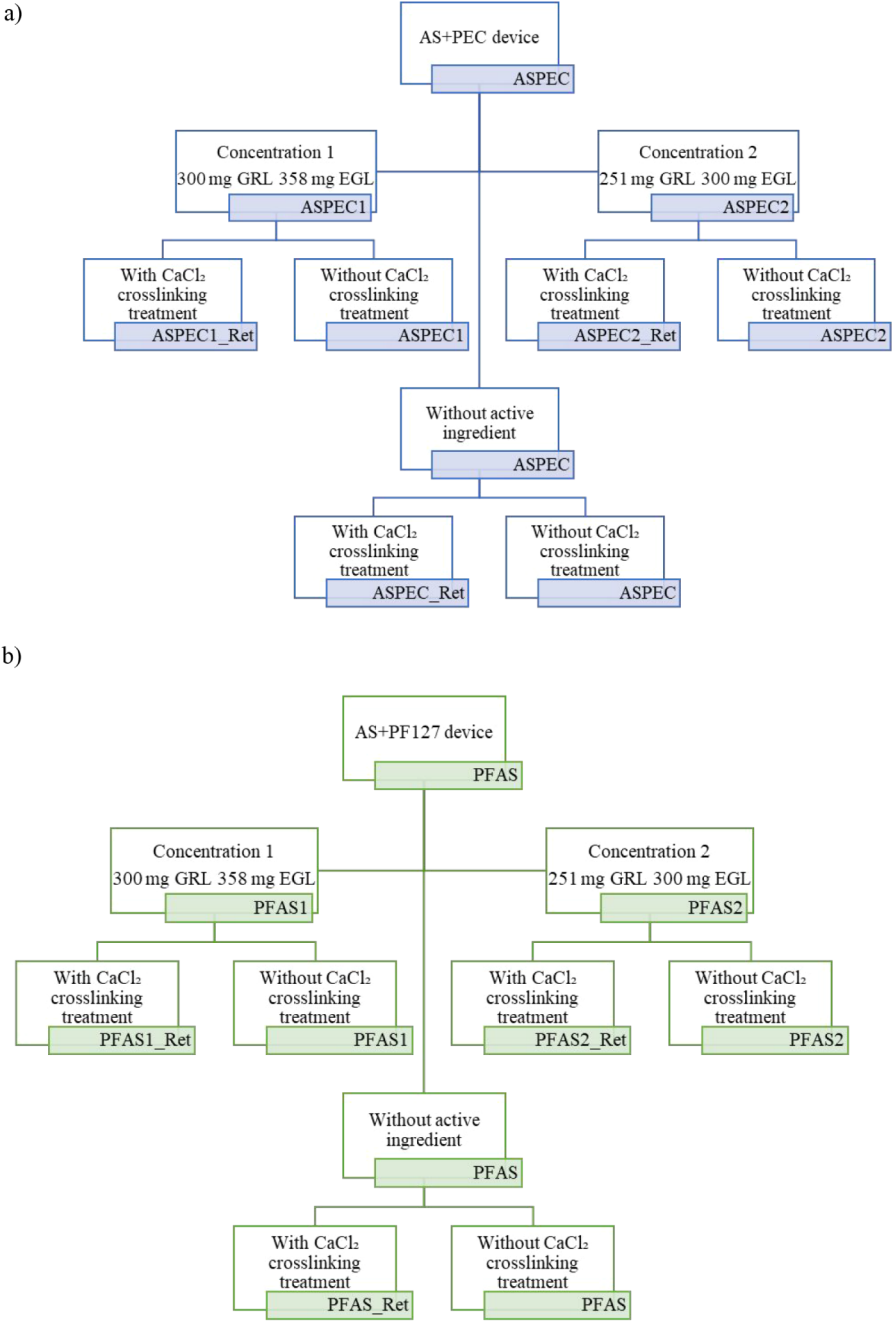
Schematic representation of the formulations tested for evaluating biological activity against the agricultural pest *Bemisia tabaci* (whitefly). Two carrier systems were investigated: (a) ASPEC (soluble starch combined with polyelectrolyte complexes (PECs)) and (b) PFAS (soluble starch combined with Pluronic F-127). Each system was tested using two concentrations of active ingredients (Concentration 1:300 mg GRL + 358 mg EGL; Concentration 2:251 mg GRL + 300 mg EGL), with and without CaCl_2_ ionic cross-linking. Controls without active ingredients were also included under both cross-linking conditions. The final acronyms denote each specific formulation used in the bioassays.

Throughout the printing process, both the deposition quality and structural stability were monitored from the extrusion to postprinting stages to assess the printability and prototype integrity. Figure 2 presents representative images of the prototypes during different stages of the process.

**Figure 2:**
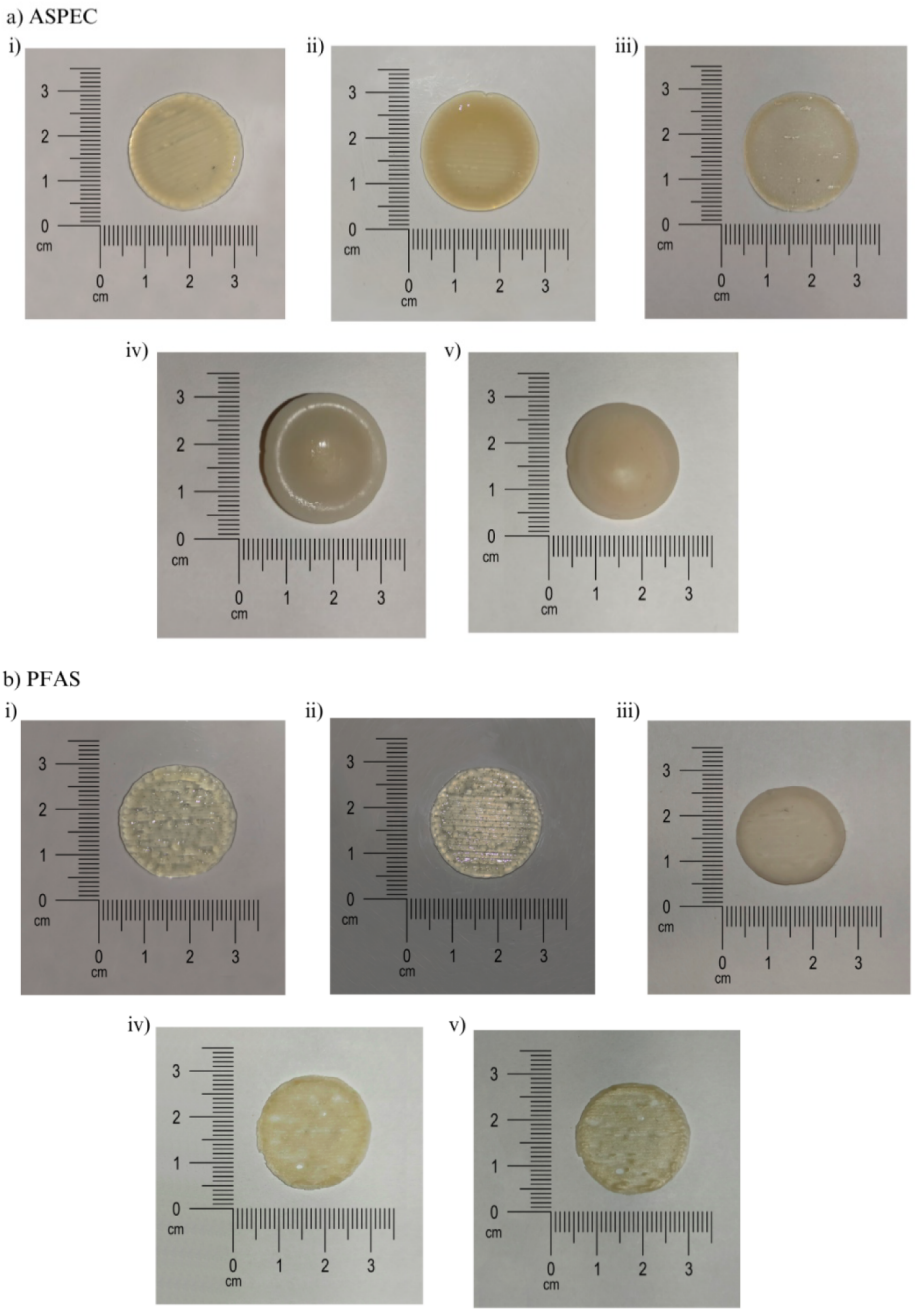
This figure presents 3D-printed prototypes of the ASPEC system composed of soluble starch and polyelectrolyte complexes under different experimental conditions. Image (i) shows the freshly printed disk immediately after extrusion. Images (ii) and (iii) display the same type of disk after ionic cross-linking with CaCl_2_ applied to side 1 and side 2, respectively, illustrating changes in surface texture and opacity. Images (iv) and (v) depict non-cross-linked disks viewed from side 1 and side 2, respectively, serving as controls. The photographs emphasize the influence of cross-linking on the physical characteristics of the biopolymeric structures, particularly regarding dimensional stability, shape retention, and surface uniformity. The scale bar indicates that each prototype is approximately 2.5 cm in diameter.

The PEC-based prototypes exhibited greater mechanical resistance and structural robustness, although they required longer drying periods regardless of the cross-linking treatment. After the mixture was dried, a noticeable size reduction was observed; however, the final structures remained rigid and easy to handle. This shrinkage behavior is attributed to polymer chain contraction following CaCl_2_-induced cross-linking,^9^ which is associated with water loss from the hydrogel network and results in reduced print volume.

In contrast, the PF127-based ink exhibited greater initial fluidity and a tendency for bubble formation, which was treated by centrifugation for 10 min at 1500 rpm until homogenization was reached. The prototypes printed with this ink exhibited greater ease of spreading during printing, resulting in a slight deformation of the structures. However, after drying and cross-linking, the samples exhibited consistency and firmness. This behavior is consistent with previous reports describing PF127 formulations as having a pasty consistency that facilitates extrusion but may compromise the initial shape fidelity.^24^

Cross-linking treatment with CaCl_2_ also caused changes in the color of the prototypes. With the use of PEC, the prototypes presented a yellowish coloration, similar to that reported in other studies.^9^ At the same time, the PF127 ink exhibited a whitish tone, suggesting that the color changes were related to the composition of the materials used.

Additionally, cross-linking improved the mechanical flexibility of both prototypes, reducing brittleness and enhancing their handling properties. Clear differences in surface texture were observed between the cross-linked and non-cross-linked prototypes, irrespective of the polymer system, with cross-linked devices showing a denser and more cohesive structure, indicating a reinforced polymer network.^25^

The selection of these formulations aimed to demonstrate the potential of 3D printing to optimize prototype design, enabling time savings and greater precision in defining dimensions and geometries. In this context, the analysis of printing parameters, including processing time, extrusion efficiency, and handling feasibility, justified the selection and combination of the PEC- and PF127-based inks employed in this study.

Thus, 3D printing technology emerges as a promising approach for sustainable management applications, as it enables the use of different polymeric matrices and the evaluation of interactions between these formulations and distinct active ingredients such as nanomaterials and essential oils.

### 3.3 Rheological Properties

To compare the effect of the tip on the structure of the material, rheological measurements were performed before and after the application of the tip to understand the rheological and mechanical properties of the hydrogels. No changes were observed in the structure of the samples (Table 4S).

Thus, the PEC sample shows an increase in the *G*′/*G*″ ratio and viscosity from 40 °C, and it was observed that, after the measurements, the material became more solid. In the PF127 sample, the material was more structured, with its transformation from solution to a gel state occurring at approximately 14 °C due to the presence of the compound (Figure 3S).

Regarding the consistency of the samples, after passing through a tip at 25 °C, they demonstrate that the presence of PEC can promote greater structuring and rigidity in the system, with high viscosity and consistency values, in addition to greater stability, with a *G*′/*G*″ ratio >1.

Regarding spreadability, the samples presented numerical values between 0.23 and 0.66, resulting in greater spreadability for the prototype with PEC, owing to the presence of PF127.

The biomaterial for printing must exhibit suitable viscosity conditions to enable controlled extrusion through the nozzles and to promote drying within a short period after printing.^28^ The materials’ viscosities exhibit a pattern similar to that observed under temperature variation. Thus, the PF127-containing sample had a higher viscosity for PEC.

The good rheological and mechanical properties observed are primarily attributed to the fact that both polymeric compositions provide sufficient internal space, thereby increasing the hydrogels’ cross-linking density,^14^ ensuring advantages in improving printing capacity and accuracy.

However, encapsulated cells have a greater impact on the mechanical properties of the printed prototype than air bubbles because the latter do not disrupt the device’s continuity.^9^ Consequently, additional rheological analyses are essential to understanding the interactions between the formulation and hydrogels.

### 3.4 Scanning Electron Microscopy *(*SEM) and Atomic Force Microscopy (AFM)

The morphological characterization of the 3D-printed hydrogels was performed by using SEM and AFM to evaluate their internal architecture and surface topography. These characteristics are key indicators of the physicochemical behavior and potential biological activity of the prototypes.^14^ The SEM analysis (Figure 3(i) and (ii)) revealed distinct differences between the PEC- and PF127-based prototypes. The PFAS prototypes exhibited a highly porous internal structure with larger pore sizes and greater spacing, indicating a more fluid initial ink and associated bubble formation during extrusion. In contrast, the ASPEC prototypes exhibited a denser, more homogeneous internal structure characterized by smaller pores, which contributed to the improved mechanical strength and rigidity.

**Figure 3:**
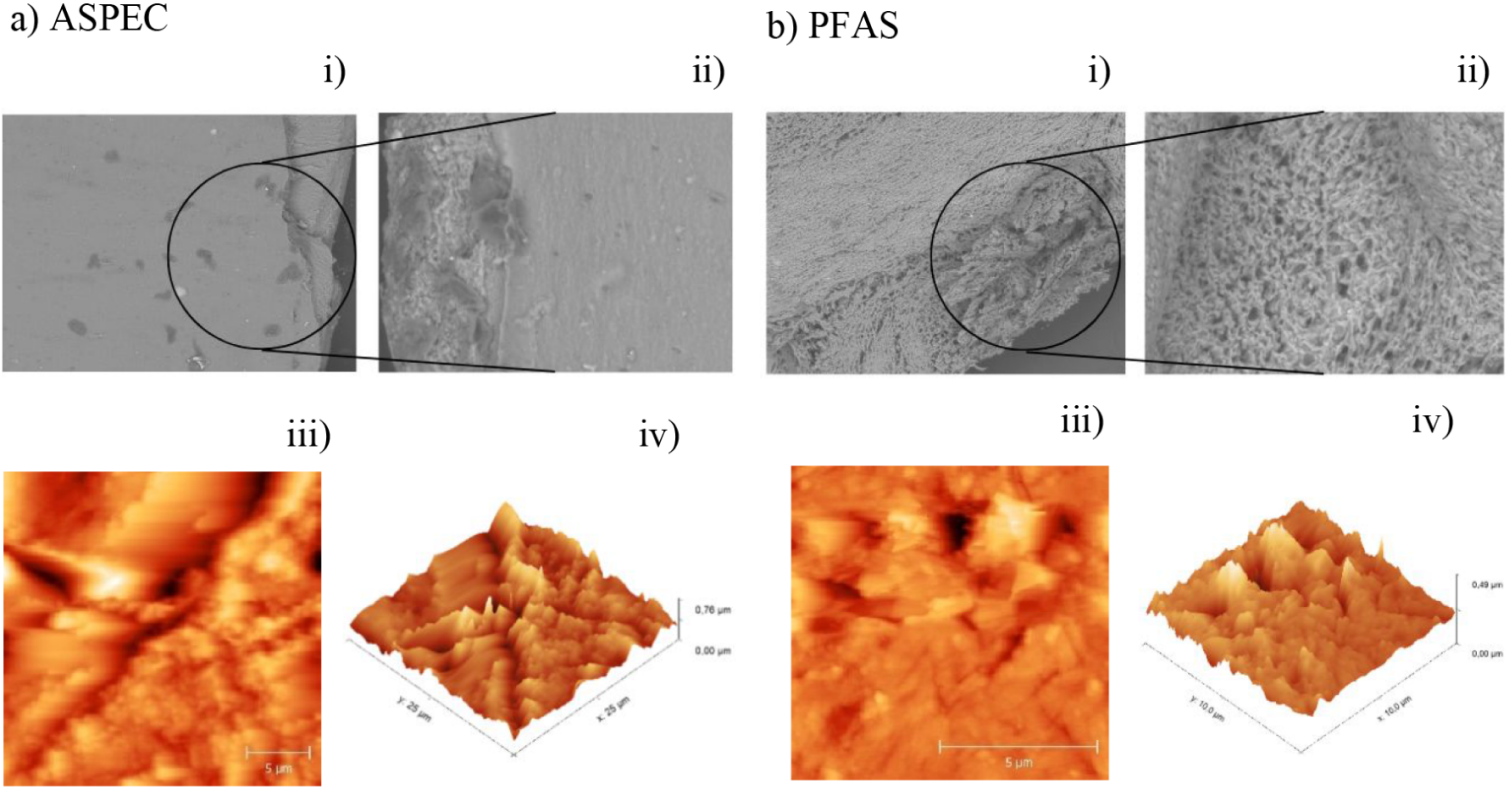
This figure compares the surface morphology of the ASPEC (a) and PFAS (b) hydrogel formulations by using complementary microscopy techniques. Scanning electron microscopy (SEM) images are presented at two magnifications: (i) 300×, showing general surface features, and (ii) 1000×, highlighting finer structural details. In both systems, the SEM images reveal distinct topographical differences related to the type of prototype used. Additionally, AFM analyses are shown in images (iii) and (iv), corresponding to 2D and 3D surface maps of the same scanned area, respectively. The AFM data confirm the presence of nanoscale surface heterogeneity and structural patterns unique to each hydrogel formulation, providing insights into their physicochemical properties and potential influence on biological interactions.

These observations are consistent with the literature, where PF127-based hydrogels are known to generate interconnected porous networks that enhance cross-linking density while maintaining flexibility.^14^ Moreover, the pectin-rich formulations tend to form more compact structures with superior mechanical integrity.

The AFM surface analysis corroborated the SEM findings, showing rough surfaces with evident topographic variations (Figure 3(iii) and (iv)). Both formulations presented localized height peaks and depressions; however, PFAS samples exhibited significantly greater surface irregularity and height variation, as evidenced by higher RMS and average roughness values (40.63 nm RMS for PFAS vs 76.99 nm RMS for ASPEC) and maximum height differences (493.9 nm for PFAS vs 763.7 nm for ASPEC). Despite the PEC samples’ larger maximum heights, the PFAS exhibited more discontinuous, uneven surface features at the micrometric scale.

These combined results indicate that, while PF127 hydrogels generate more porous and irregular microstructures, ASPEC devices exhibit smoother and more compact structures with potentially superior mechanical resistance.

It is essential to note that the SEM images were obtained from cross-linked samples, whereas AFM measurements were performed on non-cross-linked prototypes to minimize artifacts arising from height discontinuities introduced by the CaCl_2_ treatment. This methodological distinction did not compromise the comparative analysis, as both techniques consistently revealed the intrinsic morphological characteristics of each ink system.

In conclusion, the choice of biopolymer influenced the internal and surface morphologies of the 3D-printed devices. However, these differences did not translate into significant variations in biological performance, as confirmed by the bioassay results discussed later.

### 3.5 Characterization by Fourier Transform Infrared Spectroscopy

Analysis of the FTIR spectra of the hydrogels and the formulation provided insights into interactions between the ink components and the formulation elements. In the spectrum of zein, characteristic bands are observed, such as CH_2_ (3800 cm^–1^), CO bonds (1650 cm^–1^ and 1100 cm^–1^), and NH (1300 cm^–1^), typical of proteins and amino acids.^18^ Similarly, these compounds appear throughout the spectrum of the actives and Pluronic, except for the OH band (3250 cm^– 1^), which presents as a differential (Figure 4).

**Figure 4:**
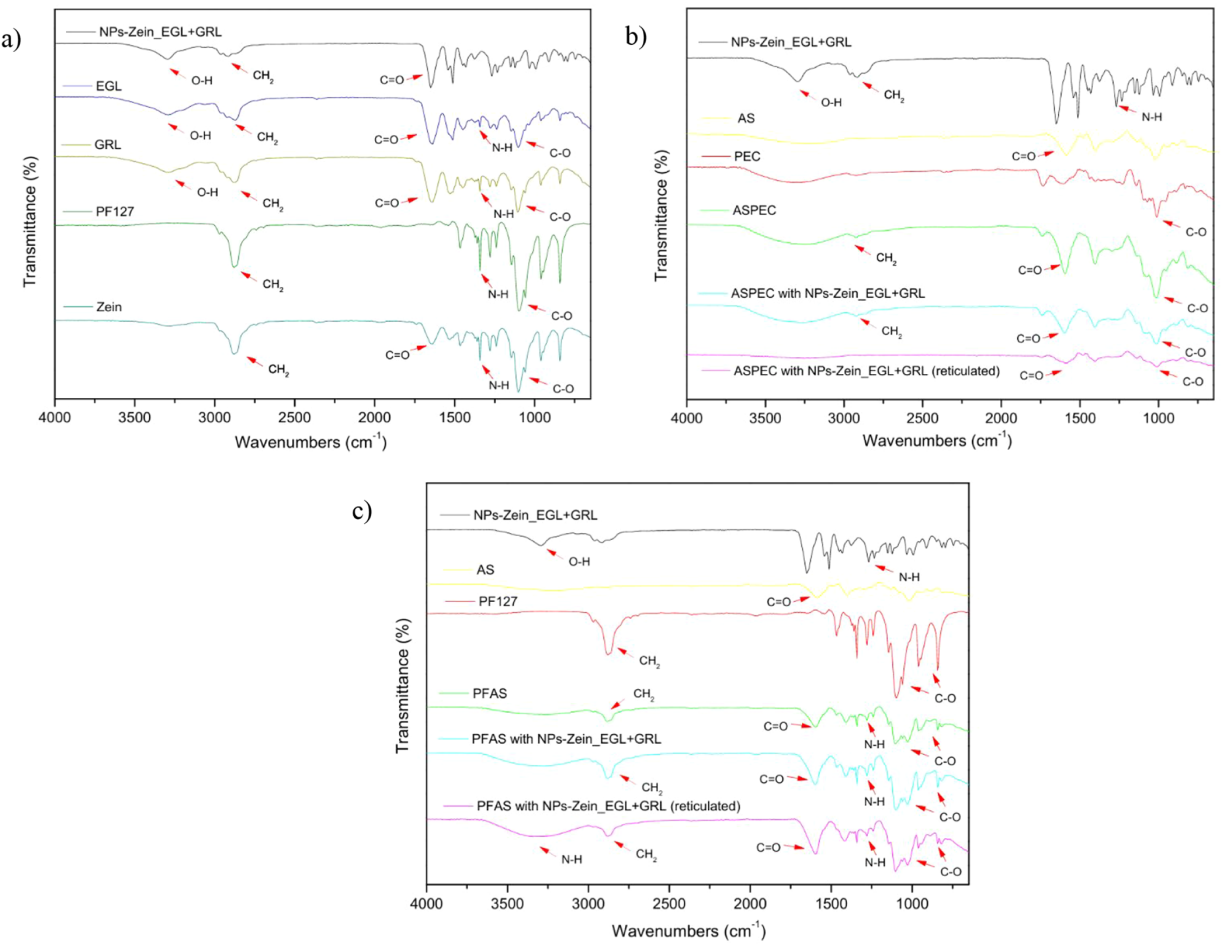
Fourier transform infrared spectroscopy (FTIR) spectra of the individual components, nanoparticle formulations, and 3D-printed matrices are shown to evaluate chemical interactions and confirm the presence of functional groups. Panel (a) presents spectra of the isolated components (Zein, EGL, GRL, and PF127) and the assembled nanoparticle formulation (NPs-Zein_EGL-GRL), identifying characteristic absorption bands related to the O–H, C–H, C=O, and C–O vibrations. Panel (b) shows spectra for the ASPEC prototype and its individual components (AS and PEC), as well as the incorporation of nanoparticles, both in non-cross-linked and CaCl_2_-cross-linked forms, indicating successful integration and possible intermolecular interactions. Panel (c) displays the corresponding data for the PFAS system, comparing the base components (AS and PF127) with the nanoparticle-loaded matrices. The spectral shifts and appearance of new peaks provide evidence of physical entrapment or chemical interactions between the device and nanoparticles in both systems.

In the spectra of the nanoparticles containing the active ingredient, the characteristic bands of each component were identified, indicating an overlap of Zein, PF127, EGL, and GRL, which is consistent with the encapsulation of the active ingredient.

The leading bands observed in the hydrogels were similar to those found in the nanoparticles (Figure 4b and c), confirming the consistency between the compounds present in the formulation and in the ink. Furthermore, when analyzing the hydrogels treated with cross-linking, the spectrum showed some changes, such as a decrease in the detected bands and peak shifts, indicative of interactions with the chemical compounds used in the treatment.^26^

FTIR analysis was performed to investigate the possible interactions between the nanoparticles and the active ingredient, as well as the samples obtained via 3D printing, thereby confirming the structure and effectiveness of the active ingredient encapsulation.

### 3.6 Assessment of Biological Activity

This study aimed to investigate the interaction between the developed prototypes and the behavior of the evaluated agricultural pests to assess their effectiveness and explore the potential applications of this technology in pest management. The biological activity assessment, presented graphically in Figures 5 and 6, was based on the application of the prototypes described in Figure 1. These results quantitatively demonstrated insect attraction per replicate under different experimental conditions as well as the attraction rate associated with the tested devices.

**Figure 5:**
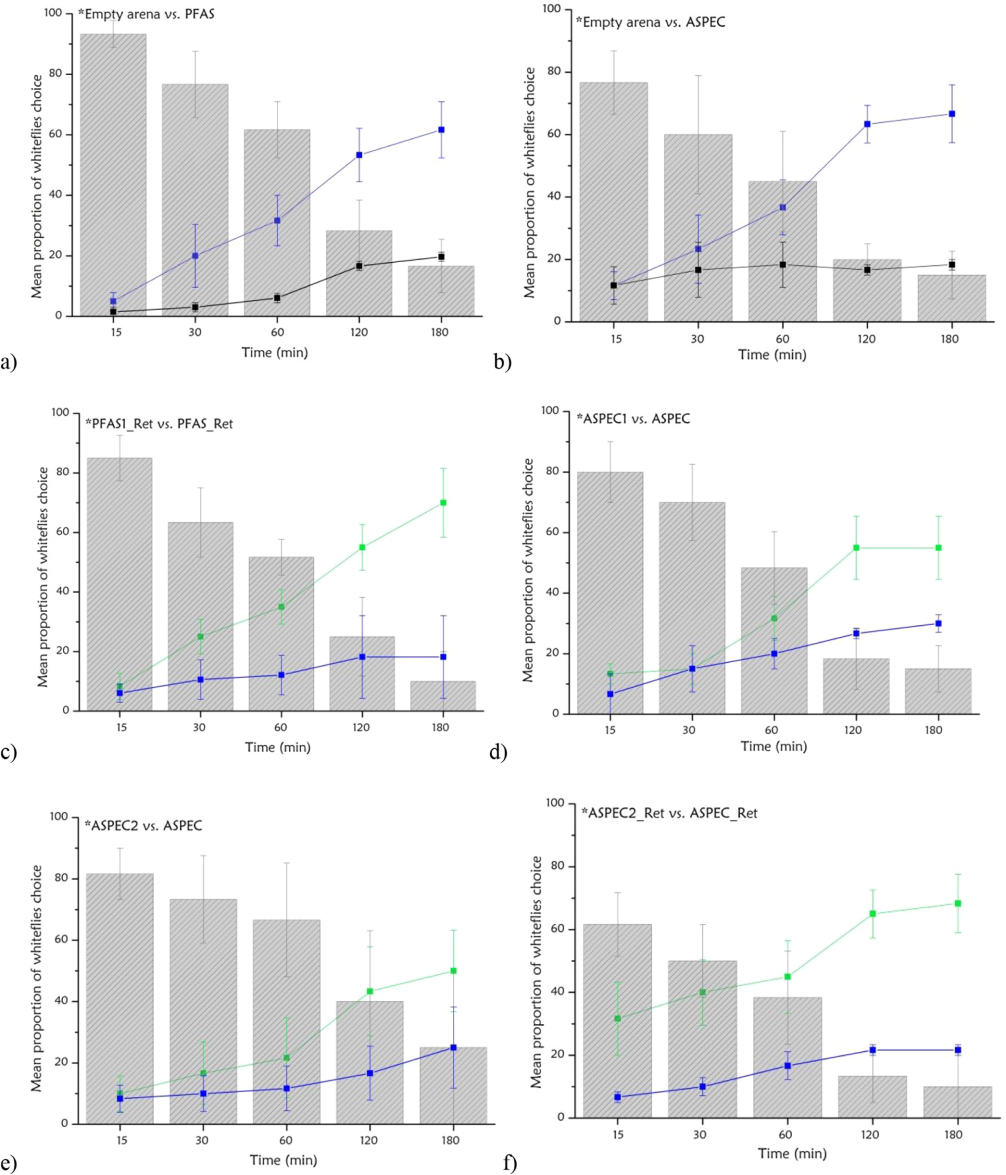
Significant bioassay combinations: (a) Empty arena vs PFAS (χ^2^ = 18.137, *p* = 0.0001), (b) Empty arena vs ASPEC (χ^2^ = 26.735, *p* = 0.0001), (c) PFAS1_Ret vs PFAS_Ret (χ^2^ = 28.316, *p* = 0.0001), (d) ASPEC1 vs ASPEC (χ^2^ = 6.684, *p* = 0.0097), (e) ASPEC2 vs ASPEC (χ^2^ = 6.969, *p* = 0.0083), (f) ASPEC2_Ret vs ASPEC_Ret (χ^2^ = 24.545, *p* = 0.0001). Mean proportion of whiteflies choosing each stimulus over time (15, 30, 60, 120, and 180 min) in two-choice bioassays (*n* = 3). Treatments include control devices (blue), active devices (green), and empty arenas (black), with insects showing no defined choice represented by gray bars. Asterisks (*) at 180 min indicate statistically significant differences in the number of insects between stimulus pairs according to chi-square tests (χ^2^), with a significance level of *p* < 0.01.

**Figure 6:**
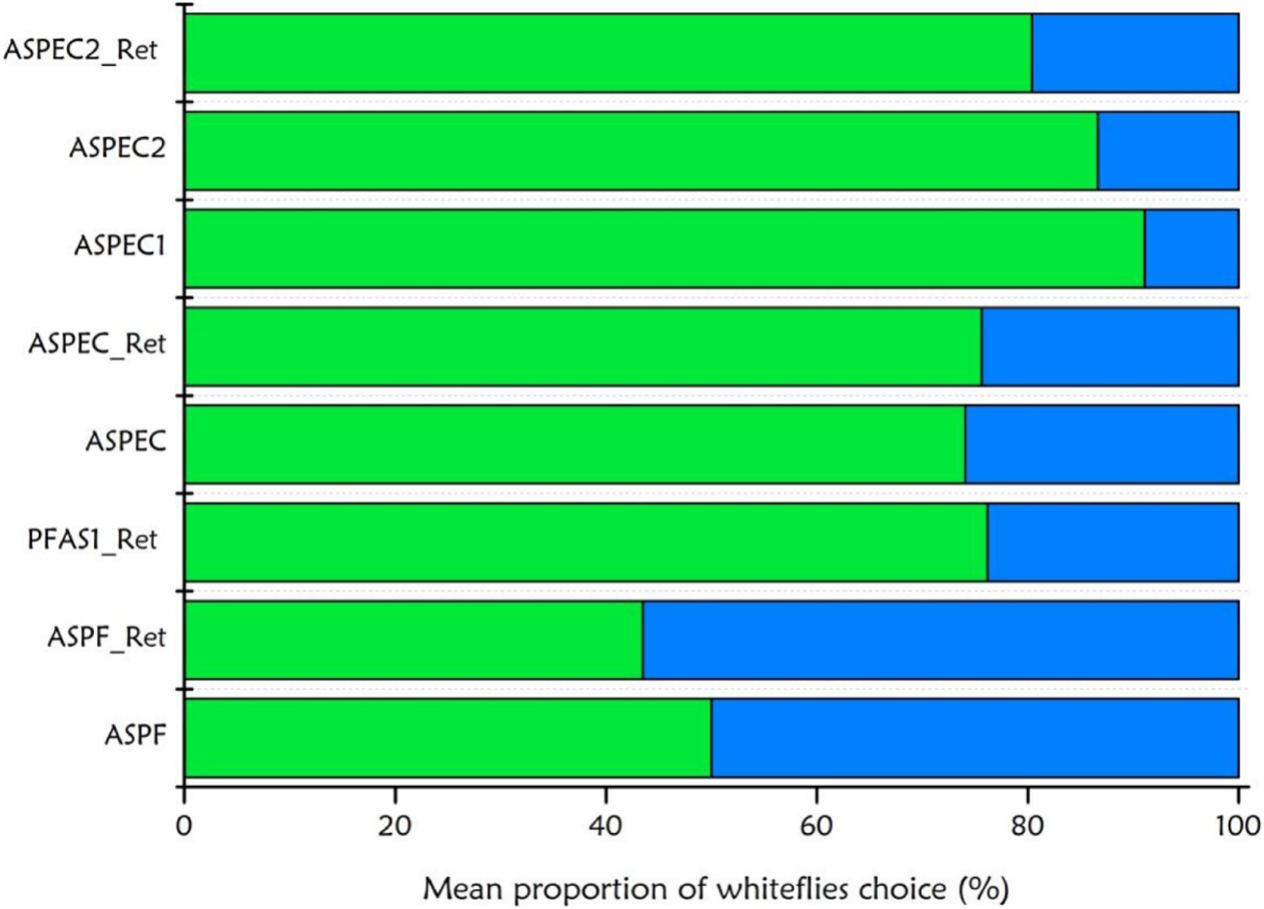
Mean proportion of *Bemisia tabaci* MEAM1 whiteflies choosing between a natural leaf (green bars) and a 3D-printed device (blue bars) in dual-choice bioassays. Each horizontal bar represents a different formulation tested, including ASPEC and PFAS systems with or without active ingredients and cross-linking treatment. The preference of whiteflies for the leaf versus the artificial device provides insights into the deterrent or attractive potential of the formulations. A lower proportion of choice for the device indicates reduced attractiveness and potential repellency, which are desirable in pest management strategies.

#### 3.6.1 Two-Choice Bioassays: Device Preference Over Time

First, groups of whiteflies (20 individuals per replicate) were introduced into arenas, where they were given a choice between environments containing the prototypes (green), control devices (blue), and empty arenas (black) to understand whether the biomaterial alone influences insect attraction compared with the presence of an active formulation.

Two-choice bioassays were conducted based on the data collected from the insects’ responses to the chosen environment, measured by counting the number of insects selecting each stimulus at time intervals of 15, 30, 60, 120, and 180 min, and performed in triplicate for each combination (*n* = 3).

In a similar trial conducted by the Brazilian Agricultural Research Corporation (Embrapa), under free-choice conditions for *Bemisia tabaci*, attractiveness was evaluated 24, 48, and 72 h after insect release by counting the number of adults present on the surface of the experimental cage.^30^ In contrast, in the present study, the evaluation was concluded at 180 min, as this period was sufficient for all individuals to establish their final positions without further movement, making the extension of the assay under controlled conditions unnecessary. It is important to emphasize that laboratory bioassays primarily aim to observe insect behavior under controlled environmental conditions, generating initial responses that inform the design of subsequent experiments simulating field conditions and guide the identification of behavioral patterns relevant to further investigation.

Statistical tests applied to the results showed that the following bioassays were significant over time (Figure 5). Asterisks (*) at 180 min indicate statistically significant differences in the number of insects between stimulus pairs according to chi-square (χ^2^) tests, with a significance threshold of *p* < 0.01.

An initial evaluation confirmed the attractiveness of both inks comprising the prototypes, as significant attraction results were observed when comparing the empty arena to the PFAS and ASPEC, with similar responses attracting between 40 and 50 insects during the analysis period.

Within environments containing prototypes formulated with the PF127 ink, the PFAS1_Ret device attracted the highest mean number of insects, with nearly 50 individuals recorded at the final 180 min interval, indicating a strong attraction response. However, only this combination yielded a statistically significant response compared to other tests involving this biomaterial, which attracted fewer insects.

In the tests conducted with the PEC prototypes, ASPEC1 and ASPEC2 stood out, each attracting nearly 30 individuals and demonstrating consistently attractive responses within 180 min. By contrast, ASPEC2_Ret demonstrated the best performance, attracting fewer than 50 individuals during the same period.

Additionally, the insect’s response to an environment without devices was tested in the presence of a tomato leaf to verify its attraction to the vegetation under study. A statistically significant result was obtained, with an attraction rate exceeding 70 insects toward the foliar environment during the study period (Figure 4S).

#### 3.6.2 Two-Choice Bioassays: Leaf Vs Device

In the second bioassay, the average proportion of whiteflies choosing between a tomato leaf and the device was measured in a two-choice scenario to assess the attractiveness of the devices. The results are shown in Figure 6, where each bar represents a significant proportion (standard error of the mean) of insects that selected the device (blue) or leaf (green) after 180 min. A total of *n* = 3 replicates were used, with 20 whiteflies per replicate.

Thus, most of the tested devices showed an attraction index above 50%, whereas others ranged between 30 and 50%, positively demonstrating the efficiency of the systems across all tested combinations.

When environments containing prototypes were compared to control environments, it was evident that prototype devices consistently attracted a greater proportion of insects. In most of the analyses, the values associated with the prototypes were consistently higher. This suggests that the presence of ink compounds plays a significant role in increasing the measured insect attraction.

In the previous assay, the PFAS device showed an attraction rate of 50%, whereas the ASPEC had a proportion exceeding 75%. Regarding the presence of active compounds in the formulas, PFAS1_Ret attracted nearly 80% of the insects, and the PEC prototypes stood out, with ASPEC1 attracting 95%, ASPEC2 attracting 90%, and ASPEC2_Ret attracting 80%.

Therefore, the best results were observed for the prototypes without cross-linking treatment in both cases. In the context of active formulation dosages, it is possible to conclude that the first combination of 300 mg of GRL and 358 mg of EGL was more effective, although the second combination showed similar responses for PEC devices.

Based on the literature, systems with slower release rates are expected to produce more pronounced attractive effects because this activity is mainly influenced by the concentration of active compounds. Furthermore, nanoencapsulated actives in hydrogels may provide longer-lasting effects than emulsified systems, facilitating more effective attraction.^26^ Thus, the behavior of the analyzed agricultural pests in relation to the prototypes can be explained by the release profile being very slow at low concentrations of essential oils, resulting in the sustained release of the compounds, as reported in the literature,^7^ which remains within a concentration range that causes attraction.

Moreover, this result may be related to the high encapsulation efficiency and the interaction of the formulation with the meshes obtained in the polymeric prototypes produced by 3D printing, which may have influenced the analysis.

Ultimately, it can be concluded that PEC hydrogels containing encapsulated botanical compounds offer superior performance, exhibiting high attraction rates while contributing to the reduced degradation of the active ingredient over time. This highlighted their potential as effective tools for sustainable pest management. Moreover, the observed sustained release profiles, high encapsulation efficiency, and interaction between the encapsulated formulation and the polymeric mesh structure generated by 3D printing likely contributed to the enhanced biological activity observed, especially in the prototypes without cross-linking treatment.

### 3.7 Comparison between the Obtained Systems

Table 1 presents the primary results obtained for the hydrogels prepared in this study. Thus, it is possible to verify the similarities and differences among the prototypes and understand their influence on the biological results obtained.

**Table 1:** Main Characteristics of the Prepared Hydrogels

	ASPEC	PFAS
Morphology	Ink with rigidity and homogeneity	Greater internal spacing, with the presence of bubbles in the structure
Viscosity	Greater spreadability	Firmness and good structuring

The PFAS hydrogel exhibited pores larger than those of the ASPEC, which may have contributed to its increased swelling capacity and potential for nanoparticle immobilization. However, this feature did not translate into enhanced biological attraction, as lower insect responsiveness was observed compared to the ASPEC.^9^ Furthermore, as previously mentioned, uniform and interconnected porous structures were observed in the PF127 hydrogels, which can enhance the cross-linking density of the device and compact its internal structure.^14^

This device also demonstrated good resistance, as indicated by rheological results, with minimal deformation after printing and a longer drying time, resulting in firm, easily produced devices. However, it showed lower biological attraction than that of the ASPEC prototypes.

The ASPEC-based devices exhibited a more homogeneous structure, characterized by lower surface roughness and greater printing flexibility. The smaller pore size of ASPEC may facilitate a more controlled and sustained release of active compounds, aligning with the significantly higher insect attraction observed in bioassays, particularly in prototypes without cross-linking treatment.^29^

However, the overall evaluation of the printed constructs revealed that both formulations demonstrated similar consistency, comparable responses to the cross-linking treatment, and a characteristic odor associated with the incorporation of essential oils.

Nevertheless, the biological results revealed a clear difference in efficacy between the prototypes. This suggests that the presence of PEC in the ink formulation may play a pivotal role in enhancing whitefly attraction, making it a promising component for the development of attractive devices for pest management. This is further supported by results from other tests using ASPEC-based devices, which consistently demonstrated a higher attraction efficiency than PFAS-based devices.

It is important to emphasize that the experiments reported in this study were conducted under controlled laboratory conditions. Moreover, because the potential selectivity of the attractant devices toward nontarget or beneficial insects has not yet been fully assessed, further field studies are required to clarify this aspect. Therefore, before large-scale adoption, additional experiments under real field conditions are recommended to more robustly elucidate the practical applicability, performance, and selectivity of the proposed devices.

## 4 Conclusions

This study demonstrated the successful development of 3D-printed hydrogel prototypes incorporating essential oils encapsulated in zein nanoparticles, achieving excellent physicochemical stability with encapsulation efficiencies approaching 100%, and maintaining structural integrity for at least 60 days. The combination of biopolymers (AS, PEC, and PF127) with a slow-release nanoencapsulated formulation allowed the generation of consistent prototypes with good mechanical support and reproducible printability.

Biological assays confirmed the attraction potential of these systems for *Bemisia tabaci* using pectin-based devices, particularly the ASPEC1 prototype, which attracted nearly 100% of insects within 180 min in two-choice bioassays. This strong attractiveness is linked to the prototypes’ controlled-release behavior, driven by their compact, homogeneous internal structures.

These results underscore the potential of nanoencapsulated essential oils combined with 3D printing as an effective tool for insect attraction, paving the way for the development of sustainable lure-and-trap systems. Furthermore, the prototypes offer advantages for reducing active ingredient degradation and present a promising strategy for integrated pest management programs aimed at minimizing the use of synthetic pesticides. However, it should be noted that the present study requires additional experimentation to elucidate its practical implications and to translate it to real field conditions, since the results were generated under controlled laboratory settings. The application of the prototypes in greenhouse environments and open-field conditions and their integration into integrated pest management (IPM) programs represent a promising scenario; nevertheless, such approaches demand that the devices be exposed to the environmental variables inherent to these contexts, which should be addressed in future studies.

## Supplementary Material



## Notes

Tables S1–S4: Detailed composition of 3D-printing materials, printing parameters, nanoparticle physicochemical stability, and rheological properties; Figures S1–S5: Schematic of the two-choice bioassay arena, nanoparticle stability over time, rheological behavior of printable inks, whitefly behavioral bioassays, and HPLC calibration curves for geraniol and eugenol (PDF)
