# Supplementary material for: 3D-Printed Hydrogel Nanoplatforms for Sustainable Pest Control: Encapsulated Essential Oils as Biopesticides against Bemisia tabaci
Source: ACS Omega. 2026 Feb 3;11(6):9460–72. doi: 10.1021/acsomega.5c09494 (PMC12917703; doi:10.1021/acsomega.5c09494)
Supplement: Supplementary file 1 [file ao5c09494_si_001.pdf]

Supporting Information

3D-Printed Hydrogel Nanoplatforms for Sustainable Pest Control: Encapsulated essential Oils as Biopesticides Against *Bemisia tabaci*

Raphaella Beatriz Barison Secco[1]; Gabriela Patrícia Unigarro Villarreal[1]; Felipe Franco de Oliveira[1,4]; Juliana Milagres[4]; Jhones Luiz de Oliveira[1,2], Daniele Ribeiro de Araujo[3]; Leonardo Fernandes Fraceto[1*].

[1] Institute of Science and Technology of Sorocaba, São Paulo State University (UNESP), Sorocaba, São Paulo, Brazil

[2] B.Nano Technological Solutions LTDA, St Dr. Julio Prestes, São Miguel Arcanjo, São Paulo, Brazil

[3] Centro de Ciências Naturais e Humanas (CCNH), Universidade Federal do ABC (UFABC), Campus Santo André, SP, Brazil

[4] The Connecticut Agricultural Experiment Station (CAES), New Haven, Connecticut, United States

Tables S1–S4; detailed composition of 3D-printing materials, printing parameters, nanoparticle physicochemical stability, and rheological properties; Figures S1–S5; schematic of the two-choice bioassay arena, nanoparticle stability over time, rheological behavior of printable inks, whitefly behavioral bioassays, and HPLC calibration curves for geraniol and eugenol.

Table 1S. Materials used for printing 3D prototypes for applications.

	Polymeric matrix	Amount (w/v)
**Prototype 1**	Sodium alginate (AS)	15%
	Pectin (PEC)	8%
**Prototype 2**	Sodium alginate (AS)	12%
	Pluronic F-127 (PF127)	10%

Table 2S. 3D printing parameters for AS with PEC and AS with PF127 prototypes.

Parameters	Prototype 1	Prototype 2
Dimension (x,y,z) (mm)	25 x 25 x 1.2	25 x 25 x 1.2
Layer (mm)	0.3	0.3
Fill (%)	25 Grid	25 Grid
Flow (%)	130	110
First layer speed (%)	80	90

Table 3S. Average values of size, PDI, ZP, pH and EE during 60 days of control NPs-Zein and NPs-Zein_GRL+EGL.

Formulation	Size (nm)	PDI	ZP (mV)	pH	EE (%)
NPs-Zein	128 ± 22	0.16 ± 0.03	28.94 ± 3.94	3.95 ± 0.12	-
NPs-Zein_EGL+GRL	318 ± 28	0.43 ± 0.05	30.28 ± 1.47	3.42 ± 0.12	GRL 99.92 ± 0.15<br>EGL 99.96 ± 0.20

Table 4S. Rheological data of the samples at 25 °C before and after using a tip, calculated from equations 1, 2.

Sample	ASPEC		PFAS	
Parameters	No tip	With tip	No tip	With tip
G'/G''	1.05	1.08	.,40	1.37
η $(Pa.s)$	127.9	132.8	132.8	158.7
K $(Pa.s^n)$	212.8 ± 11.7	228.8 ± 11.0	217.4 ± 10.9	157.6±1.2
n	0.45±0.01	0.44±0.01	0.35±0.01	0.36±0.01
R^2	0.9945	0.9946	0.9898	0.9998
η_∞ $(Pa.s)$	11.4 ± 0.8	10.8±1.0	4.9±0.2	6.2±0.6
η_0 $(Pa.s)$	89.8 ± 2.1	78.4±2.6	95,.0±0.6	66.5±1.7
C (s)	0.054±0.003	0.050±0.003	0.053±0.001	0.054±0.003
M	1.6±0.1	1.5±0.1	1.9±0.1	1.5±0.1
R^2	0.9963	0.9932	0.9997	0.9967

Figure 1S. Schematic representation of the two-choice bioassay arena used in whitefly (*Bemisia tabaci* 1MEAM1) behavioral experiments. The setup consists of two plastic Petri dishes (100 mm diameter) connected by a 10 mm diameter plastic tube. A central opening in the tube allows for the introduction of whiteflies, positioned equidistantly (50 mm) from each stimulus source.

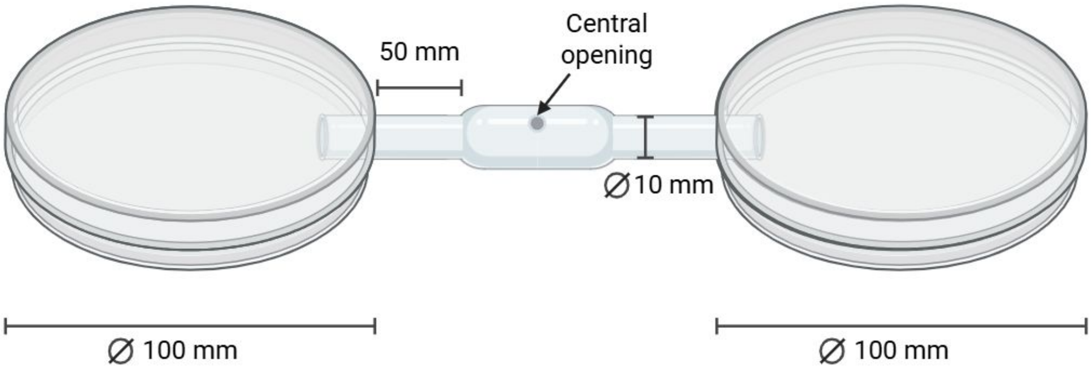

Figure 2S. Stability of (a) size (nm), (b) PDI, (c) ZP (mV), size distribution (nm) for (d) control nanoparticles and (e) active nanoparticles over 60 days, at 0, 7, 15, 30, 45, and 60 days, for NPs-Zein and NPs-Zein_EGL+GRL.

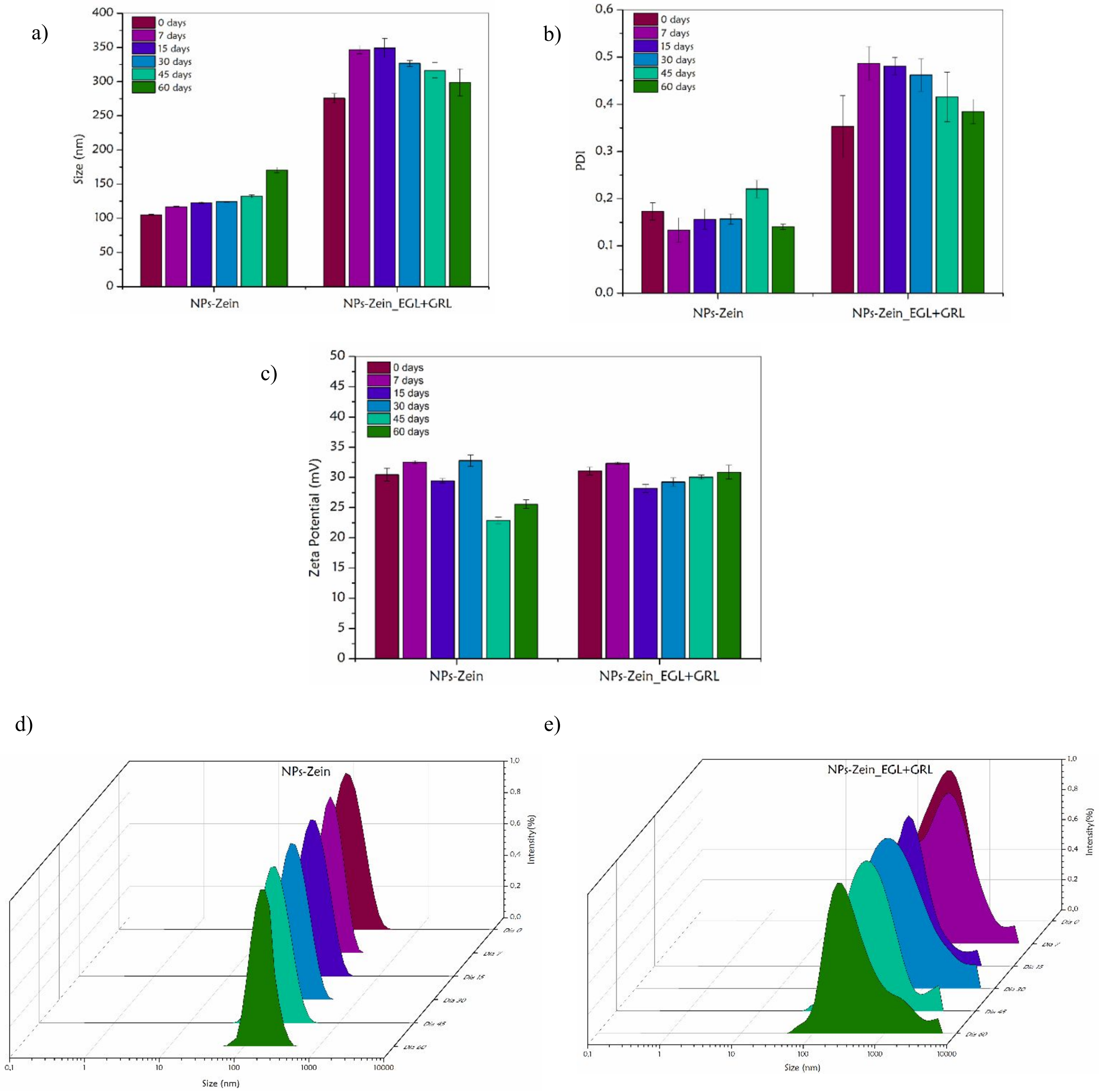

Figure 3S. Graphs showing the rheological properties of ASPEC and PFAS inks, considering the tip used for the extrusion printing process.

a) ASPEC

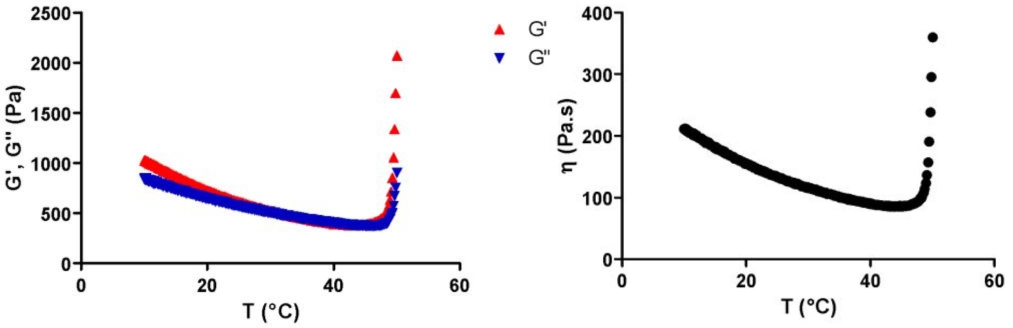

b) PFAS

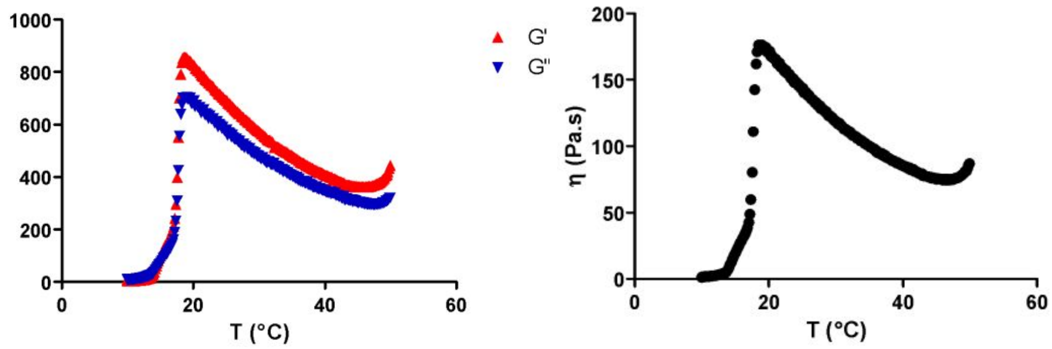

Figure 4S. Significant bioassay combinations: Leaf vs empty arena ($\chi^2 = 50.700$, $p = 0.0001$). Mean proportion of whiteflies choosing each stimulus over time (15, 30, 60, 120, and 180 min) in two-choice bioassays (n = 3). Treatments include leaf (green) and empty arenas (black), with insects showing no defined choice represented by gray bars. Asterisks (*) at 180 min indicate statistically significant differences in the number of insects between stimulus pairs according to chi-square tests (χ^2), with a significance level of $p < 0.01$.

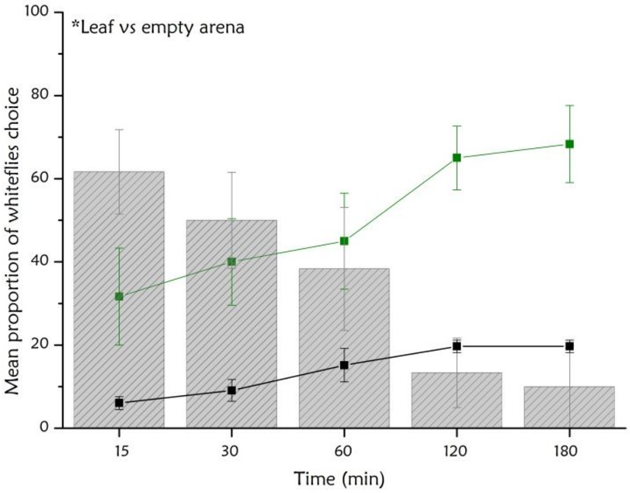

Figure 5S. Calibration curve of (a) Geraniol e (b) Eugenol. Prepared from the quantification of the concentrations of standard solutions obtained from the ultrafiltrate reading by HPLC.

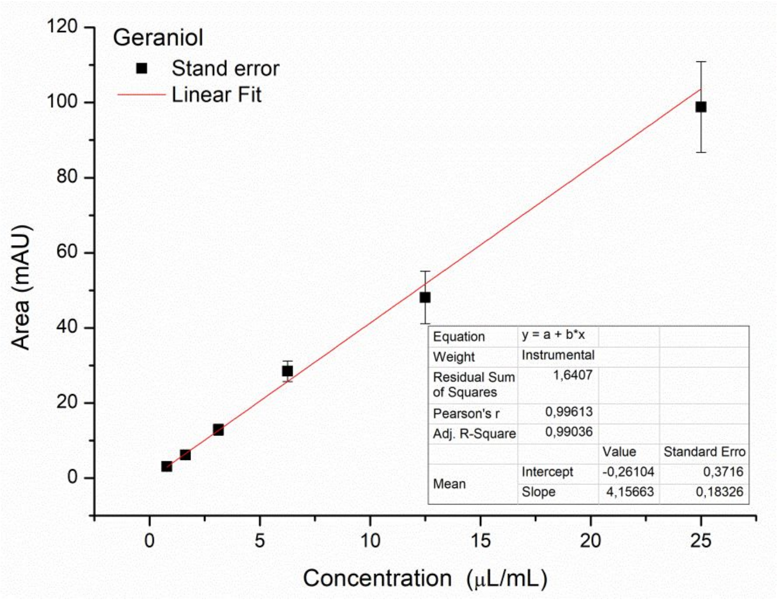

a)

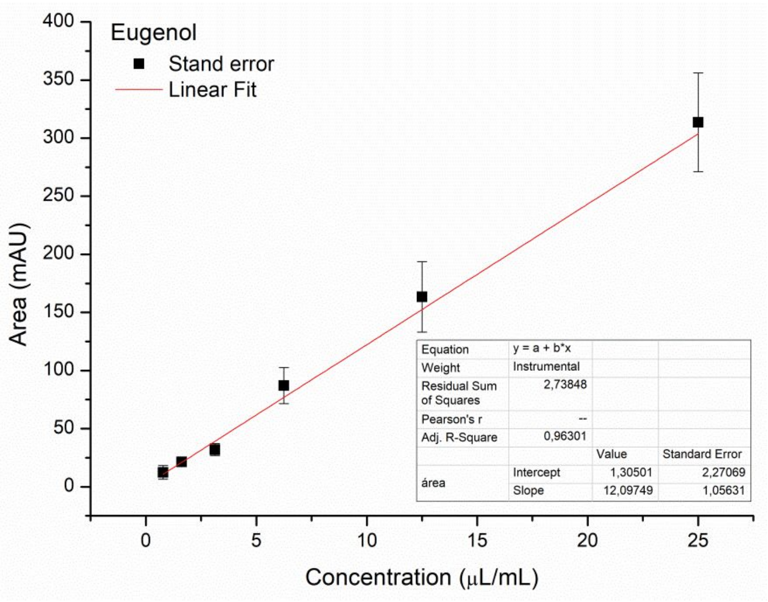

b)
